# Regulation of T-cell Receptor Gene Expression by Three-Dimensional Locus Conformation and Enhancer Function

**DOI:** 10.3390/ijms21228478

**Published:** 2020-11-11

**Authors:** Alonso Rodríguez-Caparrós, Jesús Álvarez-Santiago, María Jesús del Valle-Pastor, Carlos Suñé, Jennifer López-Ros, Cristina Hernández-Munain

**Affiliations:** Institute of Parasitology and Biomedicine “López-Neyra”—Spanish Scientific Research Council (IPBLN-CSIC), Parque Tecnológico de Ciencias de la Salud (PTS), 18016 Granada, Spain; alonsorc@ipb.csic.es (A.R.-C.); jesus_al7ez@outlook.com (J.Á.-S.); mjvp@hotmail.com (M.J.d.V.-P.); csune@ipb.csic.es (C.S.); jenni@ipb.csic.es (J.L.-R.)

**Keywords:** T-cell receptor, enhancer, transcription, V(D)J recombination, chromatin, T-cell development

## Abstract

The adaptive immune response in vertebrates depends on the expression of antigen-specific receptors in lymphocytes. T-cell receptor (TCR) gene expression is exquisitely regulated during thymocyte development to drive the generation of αβ and γδ T lymphocytes. The TCRα, TCRβ, TCRγ, and TCRδ genes exist in two different configurations, unrearranged and rearranged. A correctly rearranged configuration is required for expression of a functional TCR chain. TCRs can take the form of one of three possible heterodimers, pre-TCR, TCRαβ, or TCRγδ which drive thymocyte maturation into αβ or γδ T lymphocytes. To pass from an unrearranged to a rearranged configuration, global and local three dimensional (3D) chromatin changes must occur during thymocyte development to regulate gene segment accessibility for V(D)J recombination. During this process, enhancers play a critical role by modifying the chromatin conformation and triggering noncoding germline transcription that promotes the recruitment of the recombination machinery. The different signaling that thymocytes receive during their development controls enhancer activity. Here, we summarize the dynamics of long-distance interactions established through chromatin regulatory elements that drive transcription and V(D)J recombination and how different signaling pathways are orchestrated to regulate the activity of enhancers to precisely control TCR gene expression during T-cell maturation.

## 1. Signaling and TCR Expression during Thymocyte Development

**Figure 1:**
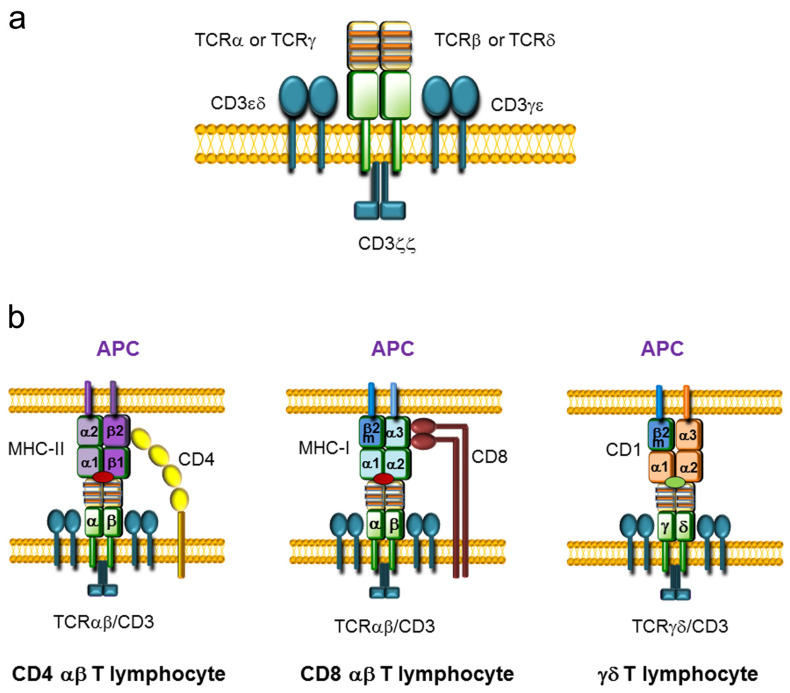
T-cell receptor (TCR) structure and TCR-antigen presenting cell (APC) interaction. (**a**) Representation of TCRαβ and TCRγδ complexes assembled with CD3 complexes on the cell membrane. TCRαβ and TCRγδ consist of two variable chains (TCRα and TCRβ or TCRγ and TCRδ, respectively) covalently bound by disulfide bridges that are associated with three CD3 dimers (CD3εδ, CD3γε, and CD3ζζ). The variable regions of the TCR chains are represented in yellow, whereas the constant (C) regions are represented in green. The three complementary determinant regions (CDRs) present within the variable regions are represented by orange stripes. The CD3 dimers are represented in blue. (**b**) Representation of the interactions between the TCR on T lymphocytes and antigen-presenting molecules on APCs. TCRαβ/CD8 or TCRαβ/CD4 present on αβ T lymphocytes and the major histocompatibility complex (MHC) loaded with a peptide (red oval) expressed on APCs are illustrated. The CD4 coreceptor is represented in yellow and the CD8 coreceptor is represented in brown. MHC molecules consist of heterodimers: MHC class I (MHC-I) is formed by an α chain and β2-microglobulin (β2m), and MHC class II (MHC-II) is formed by an α chain and a β chain. CD4 αβ T lymphocytes interact with the β2 domain of the MHC-II β chain, whereas CD8 αβ T lymphocytes interact with the α3 domain of the MHC-I α chain. The interaction between the TCRγδ and a CD1 molecule, consisting of an α chain and β2m, loaded with a lipid antigen (green oval) expressed on an APC is also represented.

During development, T lymphocytes acquire the ability to recognize foreign antigens, providing protection against diverse pathogens. Antigen recognition is driven by the expression of highly variable surface antigen receptors called T-cell receptors TCRs [1]. These heterodimeric and clonotypic receptors are responsible for recognizing fragments of antigens bound to specific molecules on presenting cells. TCRs are expressed on the cell membrane in a complex with nonpolymorphic CD3 proteins that are essential for receptor assembly and signal transduction (Figure 1a). Two different types of T lymphocytes can be distinguished based on TCR expression: the vast majority of T lymphocytes (more than 90% in humans and mice) are called αβ T cells because they express a TCRαβ composed of TCRα and TCRβ chains, whereas the remaining T lymphocytes are called γδ T cells because they express a TCRγδ composed of TCRγ and TCRδ chains. These receptors consist of a variable and a constant (C) region. The variable region is extracellular and contains three hypervariable regions, known as complementarity-determining regions (CDRs), which are involved in antigen recognition. The C region is proximal to the cell membrane and is followed by a transmembrane region and a short cytoplasmic tail.

αβ T cells are key mediators of vertebrate adaptive immunity, recognizing specific antigenic peptides loaded on major histocompatibility complex (MHC) molecules in collaboration with the CD4 or CD8 coreceptor, whereas γδ T cells play a prominent role in the recognition of antigenic lipids presented by CD1 molecules (Figure 1b) [2,3,4]. αβ T lymphocytes have the ability to respond to a wide variety of antigens due to their vast repertoire of unique TCRαβs. Although γδ T cells display very low variability in their TCRγδs compared to the TCRαβ repertoire present in αβ T cells, γδ T lymphocytes are particularly relevant in young animals, as they mediate critical responses to specific pathogens and are located at specific anatomical sites to elicit protection [4].

**Figure 2:**
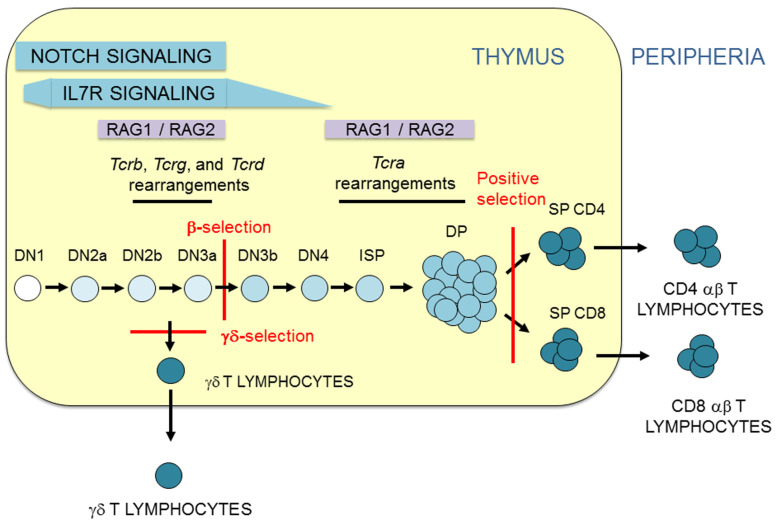
T lymphocyte development. Representation of T-cell maturation depicting the thymocyte stages and T-cell receptor (TCR) gene rearrangement. Thymus is represented as a yellow rectangle. Thymocyte and T lymphocyte populations are indicated. β-selection, γδ-selection, and positive selection are represented by red lines. The magnitude of Notch and interleukin 7 receptor (IL-7R) signaling is indicated. The two waves of recombination activating gene (RAG) proteins, RAG1 and RAG2, expression are indicated.

T cell development is a very highly controlled process in which circulating hematopoietic stem cells (HSCs) enter the thymus and differentiate to become αβ and γδ T lymphocytes [5]. During their development, thymocytes progress through a series of stages that can be distinguished by CD4 and CD8 expression: CD4^−^CD8^−^ double-negative (DN), CD8^+^ (in mice) or CD4^+^ (in humans) immature single-positive (ISP), CD4^+^CD8^+^ double-positive (DP), and CD4^+^ and CD8^+^ single-positive (SP) thymocytes (Figure 2). These populations can be further divided into different subpopulations based on the expression of specific surface markers [5,6,7]: DN1-to-DN4 thymocytes (based on CD25 and CD44 expression), DN2a and DN2b thymocytes (based on CD117 expression), DN3a and DN3b thymocytes (based on CD27 expression), and early DP (eDP) and late DP (lDP) thymocytes (based on CD71 expression). Thymocyte maturation concludes with the development of αβ or γδ T lymphocytes that circulate or reside within secondary immune organs or different anatomic locations to direct an efficient immune response.

During their maturation in the thymus, thymocytes receive different signals that direct their cellular differentiation into αβ or γδ T cells (Figure 2). The first signals that early HSCs receive upon their arrival to the thymus at the DN1 stage are mediated by Notch receptors [5]. This signaling is required for T cell commitment, which occurs when thymocytes reach the DN2a stage [8]. The process of T lymphocyte development can be divided into two phases based on Notch or TCR signaling [9]: DN1 to DN3a thymocyte development occurs in a Notch-dependent manner, whereas the development of DN3a thymocytes to αβ or γδ T lymphocytes occurs in a TCR-dependent manner. However, this division of T-cell development is not categorical because Notch signals also contribute to αβ and γδ T-cell differentiation [10,11]. Notch signaling is strong during DN1-to-DN3a thymocyte development and abruptly decreases during the DN3a to DN3b transition [6]. Coincidently with high Notch signaling, strong TCRγδ signaling can drive the development of DN2b/DN3a thymocytes into γδ T lymphocytes in a process known as γδ-selection [12]. In addition, Notch signaling plays an important role in TCRγδ expression in DN2b/DN3a thymocytes [13]. Signaling mediated by the expression of a pre-TCR formed by a TCRβ and an invariable pre-TCRα (pTα) in DN3a thymocytes is responsible for the sharp Notch signaling downregulation observed in the DN3b stage and beyond during thymocyte development [6]. Similar to its relevant role in the expression of TCRγδ in DN2b/DN3a thymocytes [13], Notch signaling is required for TCRβ and pTα expression in these cells [14,15]. Notch signaling, though present at very low levels, is required in conjunction with pre-TCR-mediated signaling for DN3a to DP thymocyte maturation in a process known as β-selection [11]. Further TCR signaling, mediated by expression of TCRαβ, is received by DP thymocytes and drives αβ T cell development in a process known as positive selection [16,17,18]. This process depends on the affinity of TCRαβ for self-peptides presented by the MHC on thymic stromal cells and concludes with the survival of only 3–5% of DP thymocytes, which then differentiate into SP thymocytes and migrate to the periphery as αβ T lymphocytes.

It is widely accepted that αβ/γδ lineage fate depends on the strength of signaling to DN3a thymocytes: thymocytes that receive low levels of signaling through the pre-TCR during β-selection develop into αβ T cells, whereas those that receive strong signals through the TCRγδ develop into γδ T cells [12]. In this context, Notch signaling contributes to the determination of αβ/γδ fate by modulating the intensity of the signals received during thymocyte development [10]. However, opposite outputs are observed in humans and mice: exogenous strong Notch signaling in murine uncommitted T cell progenitors promotes αβ T cell differentiation, whereas it promotes γδ T cell differentiation in human cells [10]. The controversial and seemingly opposite roles of exogenous Notch signaling in the development of αβ and γδ T lymphocytes in humans and mice appear to be a consequence of the cell stage at which these signals are received in each case [10]. In addition to Notch and TCR signals, signaling mediated by the interleukin 7 receptor (IL-7R), which decays during the transition from DN4 to DP thymocytes (Figure 2), is essential for cell survival and maturation [19].

## 2. TCR Genes

**Figure 3:**
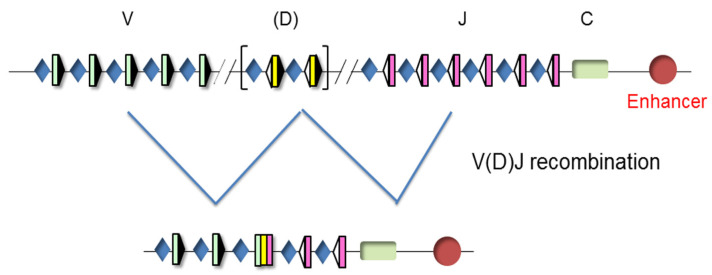
Unrearranged and rearranged T-cell receptor (TCR) gene structure. Variable (V), diversity (D), and joining (J) gene segments are indicated by green, yellow, and pink rectangles, respectively. Recombination signal sequences (RSSs) are represented by triangles adjacent to the V, D, and J gene segments (black triangles indicate RSSs with a 23 bp spacer and white triangles indicate RSSs with a 12 bp spacer). The constant (C) region is represented as a green rectangle. The enhancer is represented as a red circle and the promoters associated with the V, D, and J gene segments are represented by blue diamonds. V(D)J rearrangements are represented by blue lines.

The TCR genes can be traced back to our jawed vertebrate ancestors. These genes are expressed in a clonally diverse repertoire and generate an adaptive immune response that recognizes and repels an infection by pathogen invaders [20]. These genes are composed of multiple dispersed gene segments, including the variable (V), diversity (D), and joining (J) segments, that recombine during thymocyte development through a process known as V(D)J recombination, resulting in a new rearranged gene configuration and the expression of a functional protein [21,22] (Figure 3).

**Figure 4:**
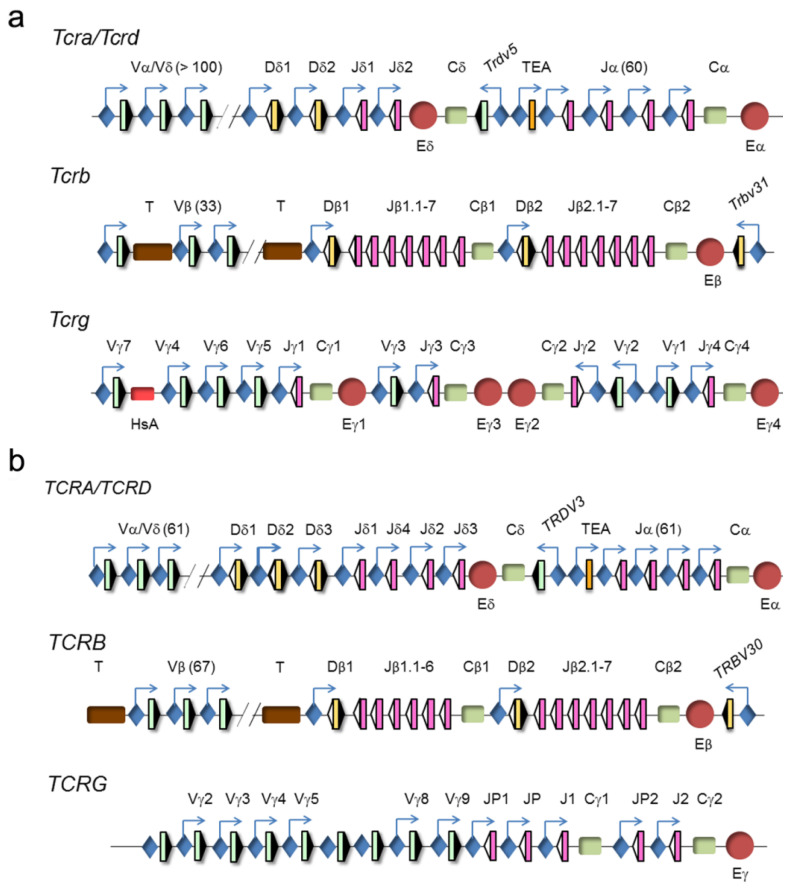
Genomic representation of mouse and human T-cell receptor (TCR) loci. (**a**) Murine and (**b**) human TCRα/TCRδ, TCRγ, and TCRβ loci are represented. Variable (V), diversity (D), and joining (J) segments are indicated by green, yellow, and pink rectangles, respectively. Recombination signal sequences (RSSs) are represented by triangles adjacent to the V, D, and J gene segments (black: RSSs with a 12-bp spacer and white: RSSs with a 23-bp spacer). Constant (C) regions are represented as green rectangles. The T early α exon (TEA) is represented as an orange rectangle. Trypsinogen genes (T) are represented as brown rectangles. Enhancers are represented by red circles and promoters by blue diamonds, respectively. Germline transcription is indicated by blue arrows.

The four TCR genes are organized within three loci, TCRα/TCRδ, TCRβ, and TCRγ (www.imtg.org) [23]. A genomic representation of mouse and human loci is shown (Figure 4).

The clustering of the TCRα and TCRδ genes at a single genomic locus, TCRα/TCRδ, is conserved in all vertebrates down to teleost fish, indicating that both genes have been linked for more than 400 million years [24]. In mammals and birds, this locus presents a nested structure with an evolutionary history of approximately 150 million years [25]. This structure prevents the coexistence of rearrangements at both genes because VαJα rearrangements result in deletion of the TCRδ gene as an extrachromosomal circle [26]. However, this organization is dispensable for the correct temporal regulation of TCRδ and TCRα gene expression and generation of αβ and γδ T lymphocytes [27]. In mice and humans, the TCRα/TCRδ locus is found between the olfactory receptor genes and *Dad1/DAD1* on chromosome 14 (www.imtg.org). The TCRα/TCRδ locus spans approximately 1500–1800 kb in mice, depending on the strain, and approximately 950 kb in humans [23,28]. The last 100 kb at the 3′-end of the locus includes the Dδ gene segments (2 in mice and 3 in humans) and Jδ gene segments (2 in mice and 4 in humans), the four exons of the TCRδ constant region (Cδ), one Vδ gene segment in an inverted orientation with respect to all the other locus gene segments (denoted *Trdv5* in mice and *TRDV3* in humans), the Jα gene segments (60 in mice and 61 in humans, of them, 43 in mice and 51 in humans are functional), and the four exons of the TCRα constant region (Cα). The large genomic region at the 5′-end includes the remaining Vα/Vδ gene segments (138 total and 117 functional in the C57bl6 mouse strain, 104 total and 88 functional in the 129 mouse strain, and 61 total and 46 functional in humans). The main difference between the mouse and human loci is the absence of recent duplications of the V gene segments in humans [29]. Among the V gene segments, most are considered Vα because they only rearrange with Jα gene segments, a few are considered Vδ because they only rearrange with Dδ gene segments, and others are considered Vα/δ because they can recombine with both Jα and Dδ gene segments. Interestingly, the V(D)J rearrangement programs at the TCRδ and TCRα genes are divergent, undergoing VδDδJδ rearrangements in DN2a/DN3a thymocytes and VαJα rearrangements in DP thymocytes [23,26,28].

The TCRγ locus spans a region of 200 kb at chromosome 13 in mice and 156 kb at chromosome 7 in humans. This locus consists of several clusters of Vγ and Jγ gene segments and a constant region (Cγ) of three to five exons [23,30]. The murine locus contains four γ1-γ4 clusters, of which only clusters γ1, γ2, and γ4 are functional. This locus contains a total of 8 Vγ gene segments, of which 7 are functional. The murine cluster γ1 contains 4 Vγ and 1 Jγ gene segments, cluster γ2 contains 1 Vγ and 1 Jγ gene segments, and cluster γ4 contains 2 Vγ and 1 Jγ gene segments. The murine cluster γ1 is the best-studied because its 4 Vγ gene segments rearrange in a very regulated fashion during embryo to adult development [31]. The proximal Vγ5 and Vγ6 (also known as Vγ3 and Vγ4, respectively, based on a previous nomenclature [32]) gene segments rearrange with Jγ1 in early fetal thymocytes to encode invariant TCRγ chains in the γδ T lymphocytes present at specific locations: Vγ5 γδ T cells in the epidermis and Vγ6 γδ T cells in vaginal and tongue epithelia. In adults, the more distal Vγ7 and Vγ4 (also known as Vγ5 and Vγ2, respectively [32]) gene segments can rearrange with Jγ1, along with Vγ2 (also known as Vγ1.2 [32]) to Jγ2 and Vγ1 (also known as Vγ1.1 [32]) to Jγ4, to contribute to the diverse TCRγ repertoire present in secondary immune organs, with the exception of Vγδ cells that predominate in the intestine. The human locus contains 14 Vγ gene segments, of which only 6 are functional, and two Jγ-Cγ gene clusters, including 3 Jγ gene segments in cluster γ1 and 2 Jγ gene segments in cluster γ2.

The TCRβ locus spans 670 kb of mouse chromosome 6 and 620 kb of human chromosome 7. The 5′-end 300 kb region of the murine locus includes 33 Vβ gene segments, of which 21 are functional, whereas the same region of the human locus includes 67 Vβ gene segments, of which only 46 are functional. The trypsinogen genes are interspersed between the Vβ and Dβ gene segments in both the murine and human TCRβ loci. The 3′-end ~26 kb region in mice and humans is composed of two Dβ-Jβ-Cβ clusters, with one Dβ gene segment, 6 or 7 Jβ gene segments, one constant TCRβ gene region (Cβ1 or Cβ2) with four exons, and one inverted Vβ gene segment (*Trbv31*, also known as Vβ14, in mice and *TRBV30* in humans). These two clusters are derived from a duplication event during evolution.

## 3. V(D)J Recombination Control during Thymocyte Development

**Figure 5:**
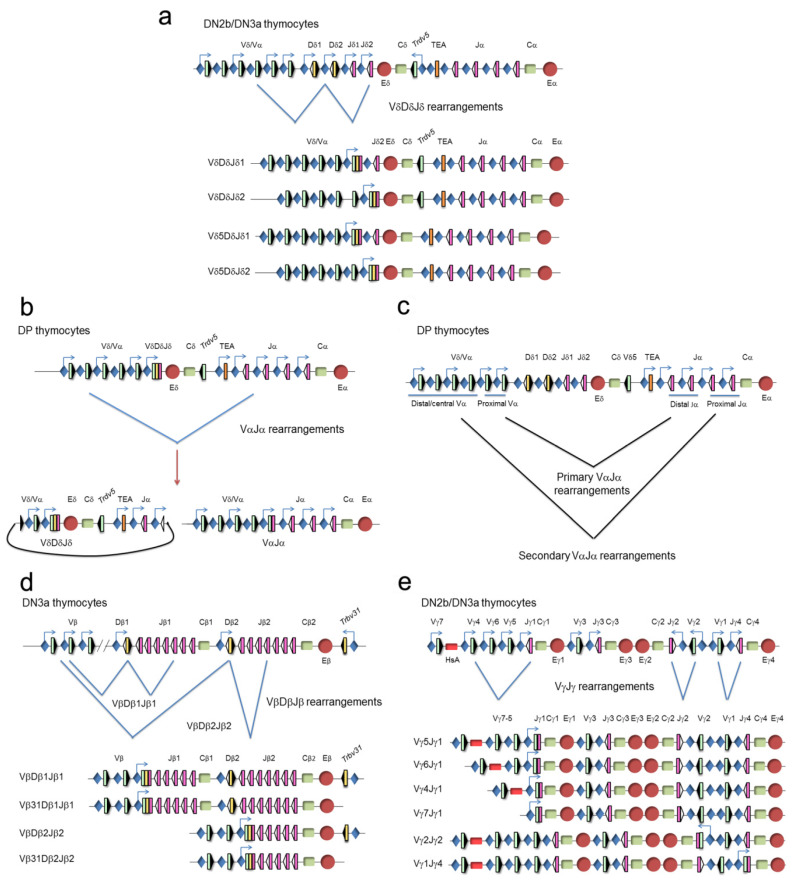
Genomic representation of the unrearranged and rearranged T-cell receptor (TCR) genes during thymocyte development. Rearrangements at the (**a**) TCRδ, (**b**,**c**) TCRα, (**d**) TCRβ, and (**e**) TCRγ genes are represented. Variable (V), diversity (D), and joining (J) gene segments are represented by green, yellow, and pink rectangles, respectively. Recombination signal sequences (RSSs) are represented by triangles adjacent to the V, D, and J gene segments (black: RSSs with a 12-bp spacer and white: RSSs with a 23-bp spacer). Constant (C) regions are represented as green rectangles. The early α exon (TEA) is represented as an orange rectangle. Trypsinogen genes (T) are represented as brown rectangles. Enhancers are represented by red circles and promoters by blue diamonds, respectively. Germline transcription is indicated by blue arrows. At the TCRδ gene (**a**), VδDδJδ rearrangements occur in double-negative 2b (DN2b)/double-negative 3a (DN3a) thymocytes, whereas VαJα rearrangements occur at the TCRα gene in double-positive (DP) thymocytes (**b**,**c**). As a consequence of a primary VαJα rearrangement (**c**), the TCRδ gene is deleted from the genome as an extrachromosomal circle (**b**). At the TCRβ locus (**d**), complete VβDβJβ rearrangements occur in DN3a thymocytes. At the TCRγ locus (**e**), VγJγ rearrangements occur in DN2b/DN3a thymocytes.

The different structures of the V, D, and J gene segments at the TCR gene loci establish that TCRδ gene expression results from VδDδJδ recombination (with possible inclusion of more than one Dδ gene segment), TCRα gene expression results from VαJα gene recombination, TCRγ gene expression results from VγJγ recombination, and TCRβ gene expression results from VβDβJβ recombination (Figure 5). Only one productive TCR gene rearrangement, determined at least in part by the specific gene segment organization at each TCR locus, is normally present in a given T cell clone. With the exception of the TCRβ gene, in which complete productive VβDβJβ rearrangements are restricted to a unique allele in a phenomenon known as allelic exclusion, both alleles of the other TCR genes can simultaneously rearrange [33]. The genetic structure of the TCRα/TCRδ locus organization dictates that one unique VδDδJδ rearrangement can occur per allele, whereas multiple VαJα recombination events can occur per allele (Figure 5a–c). At the TCRβ gene, only one VβDβJβ rearrangement can occur per allele (Figure 5d), whereas in the case of the TCRγ gene, several possible productive VγJγ rearrangements (one at each functional cluster) can occur independently per allele (Figure 5e). The possibility of successful VαJα rearrangements at the two TCRα gene alleles and VγJγ rearrangements at the various TCRγ gene clusters can lead to the expression of two different TCRα and multiple TCRγ chains in a relatively high percent of T lymphocytes; these TCRα and TCRγ chains can then pair with the same TCRβ or TCRδ chain, respectively [34,35]. Although it is estimated that approximately 30% of αβ T lymphocytes display in-frame VαJα rearrangements, only 10% of cells express dual TCRαβ probably due to the restriction imposed by the pairing competence between the two TCRα chains and the TCRβ or CD3 chains [34].

As previously mentioned, the process of V(D)J recombination at the different TCR genes is very precisely controlled during thymocyte development, allowing cell surface expression of one of the three possible TCRs: pre-TCR, TCRγδ, or TCRαβ. Developmental regulation of TCR expression depends on the timing of V(D)J recombination at the different TCR genes [23,36]. The combination of Notch, IL-7R, and pre-TCR signaling during thymocyte development controls the temporally regulated V(D)J recombination and TCR chain expression. V(D)J recombination occurs at two different times during T cell development (in DN2b/DN3a thymocytes and in DP thymocytes) due to two waves of RAG1 and RAG2 expression [37] (Figure 2). The first wave of RAG1 and RAG2 expression is responsible for TCRβ, TCRγ, and TCRδ gene rearrangements, whereas the second wave activates the TCRα gene rearrangements. Expression of the different TCRs drives specific selection processes to direct maturation of thymocytes into functional αβ or γδ lymphocytes. Successful VγJγ and VδDδJδ rearrangements in DN2b and DN3a thymocytes allow expression of a TCRγδ in these cells that drives thymocyte maturation into γδ T lymphocytes during the γδ-selection process. Successful completion of VβDβJβ gene rearrangements in DN3a thymocytes allows expression of a TCRβ chain that assembles with pTα to form a pre-TCR that drives thymocyte maturation into DP thymocytes, via the DN3b, DN4, and immature SP stages, during the β-selection process. During these processes, Notch and IL-7R signaling play very relevant roles concomitant with TCR signaling in DN2b-DN3a thymocytes. Notch signaling is required for expression of TCRγδ and the pre-TCR components TCRβ and pTα [13,14,15] and is essential for β-selection [11], whereas IL-7R signaling is required for expression of the TCRγ chain [38,39,40,41] and is essential for the generation of γδ T lymphocytes [42,43]. As a consequence of β-selection, RAG1 and RAG2 are re-expressed and VαJα rearrangements take place in DP thymocytes, allowing expression of a TCRα chain that pairs with the previously selected TCRβ chain to form a TCRαβ in these cells. Another consequence of β-selection is the stable transcriptional silencing of the rearranged TCRγ and TCRδ genes, which prevents expression of TCRγ and TCRδ chains in DP thymocytes and αβ T lymphocytes that would interfere with the normal assembly of a functional TCRαβ [44,45,46]. Silencing of TCRγ and TCRδ genes is mediated by the pre-TCR through the inhibition of Notch and IL-7R signaling [13].

## 4. Architectural Changes at the TCR Regions during Thymocyte Development

In addition to the different unrearranged and rearranged gene configurations, considerable changes in the genomic 3D architecture occur at the TCR loci during lymphocyte development in a V(D)J recombination-independent manner. These different genomic architectural conformations precede V(D)J recombination and provide a dynamically regulated chromatin architecture that allows temporally regulated V(D)J rearrangements, shaping the final TCR repertories. Global architecture of the TCR genes is determined by the formation of chromatin loops anchored through the recruitment of CCCTC-binding factor (CTCF) and cohesin to convergently oriented CTCF binding sites (CBSs) [47]. These developmentally regulated loops, together with the chromatin changes mediated by enhancer-promoter interactions at the D-J region, control the accessibility of V, (D), and J gene segments to RAG1 and RAG2 proteins by RSS scanning through a process of chromatin extrusion, establishing a molecular basis for the developmental control of V(D)J recombination [48,49]. Formation of these loops brings gene segments into spatial proximity to trigger V-to-(D)J recombination and to provide similar opportunities for the rearrangement of different V gene segments. The diverse V gene segment usage in the locus rearrangements that occur in lymphocyte precursors is likely a consequence of high heterogeneity of these long-range looping interactions due to their dynamic nature, constant assembly/disassembly, or formation of a different loop in each individual cell [47].

### 4.1. Topological Gene Changes at the TCRα/TCRδ and TCRβ V Regions

**Figure 6:**
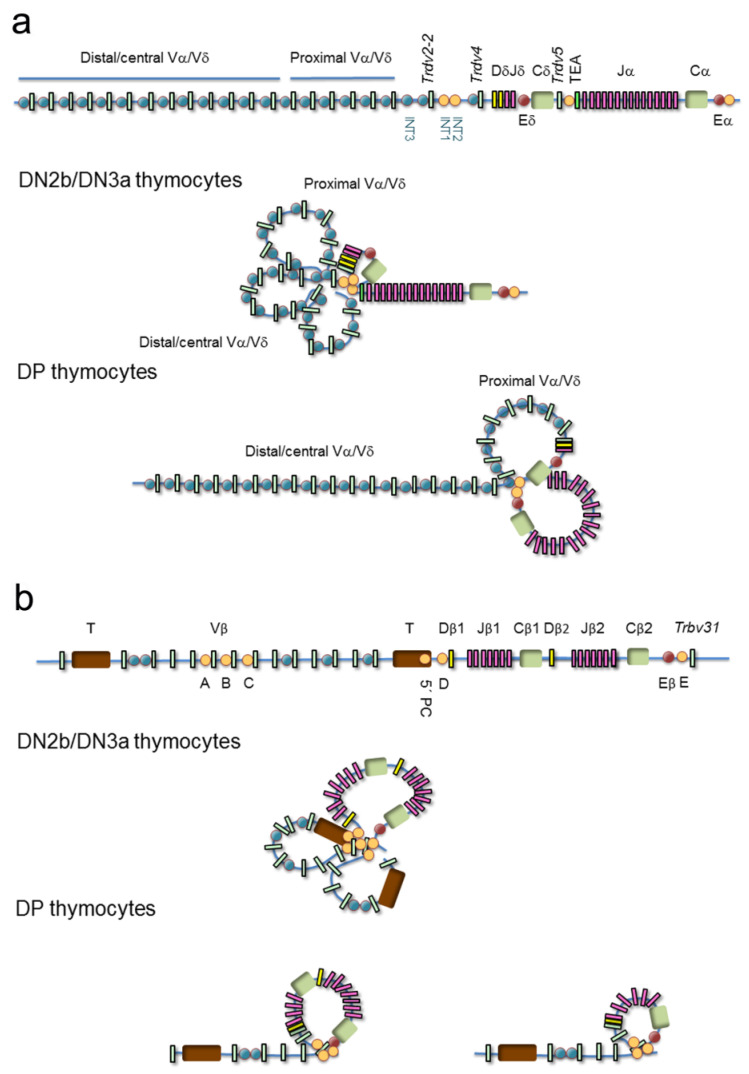
Models of chromatin loop configuration at the T-cell receptor (TCR) loci: TCRα/TCRδ and TCRβ. Variable (V), diversity (D), and joining (J) gene segments are represented by green, yellow and pink rectangles, respectively. The T early α exon (TEA) is represented as an orange rectangle. Trypsinogen genes (T) are represented by brown rectangles. Enhancers are represented by red circles. CTCF binding sites (CBSs) are represented by blue or orange circles (the orange circles indicate the most relevant CBSs involved in three-dimensional gene structure). (**a**) The TCRδ recombination center (RC) is established by a chromatin loop between INT1/2 and TEA promoter (TEAp) CBSs in double-negative 2b (DN2b)/double-negative 3a (DN3a) thymocytes, whereas the 5′ Vα/δ and Vδ gene segment region forms additional loops that result in locus contraction. In double-positive (DP) thymocytes, the TCRα RC is established by a chromatin loop between TEAp and TCRα enhancer (Eα)-adjacent CBSs, whereas additional loops are formed between the RC CBSs and CBSs associated with proximal Vα gene segments to facilitate processive VαJα rearrangements. (**b**) In DN3a thymocytes, the TCRβ RC is established by a loop between CBSs present upstream of the Dβ1 promoter and downstream of the TCRβ enhancer (Eβ), whereas additional loops are formed among the Vβ associated CBSs resulting in locus contraction, thereby facilitating the usage of distal Vβ gene segments. In DP thymocytes, the Vβ region presents an extended configuration that is involved in maintaining allelic exclusion.

Confocal microscopy has been used to describe the various genomic architectural conformations derived from chromatin loops established at the V regions of the murine TCRα/TCRδ and TCRβ loci during thymocyte development [50,51] (Figure 6). In DN3a thymocytes, the TCRα/TCRδ locus is fully contracted to allow VδDδJδ rearrangements involving Vδ gene segments distributed over 1000 kb, whereas in DP thymocytes, the 3′ end remains contracted but the 5′ end of the locus is uncompacted [50] (Figure 6a). Further analyses by chromosomal conformation capture (3C)-derived techniques have confirmed the existence of a 525-kb chromatin hub in DP thymocytes. This hub exists as a rosette structure formed by several loops of CTCF/cohesin-bound CBSs present at the proximal Vα gene segment promoters, the T early α exon (TEA) promoter (TEAp) located upstream of the Jα gene segment cluster, and the 3′ region adjacent to the TCRα enhancer (Eα) [28,47,52,53]. Formation of this chromatin hub favors the Eα-dependent activation of proximal Vα and distal Jα promoters, bringing the proximal Vα gene segments into close proximity to the most 5′ Jα gene segments, thereby promoting the ordered usage of the 3′ to 5′ Vα and 5′ to 3′ Jα gene segments in DP thymocytes necessary for processive VαJα rearrangements [52,54,55,56]. These ordered VαJα rearrangements bias the initial (primary) rearrangements to the proximal Vα gene segments, reserving a large pool of central/distal Vα gene segments for secondary rounds of VαJα recombination [50,56] (Figure 5c). This TCRα gene rearrangement strategy permits several rearrangements per allele: if a primary VαJα rearrangement in eDP thymocytes is not productive, secondary rearrangements involving more 5′ Vα gene segments and more 3′ Jα gene segments can occur during the life span of lDP thymocytes [56,57]. This mechanism assures that all lDP thymocytes express a functional TCRα chain that can pair with the previously selected TCRβ chain to form a TCRαβ, permitting cells to be positively selected. Developmentally regulated cohesin recruitment to CBS-bound CTCF within the V regions is generally accepted to be involved in the developmentally regulated topological changes observed at the TCR genes by confocal microscopy [47]; however, contraction at the TCRα/TCRδ locus was not significantly reduced upon conditional deletion of CTCF [52]. Other DNA-binding proteins may participate in this process as has been proposed for Yin-Yang protein 1 (YY1) and the B-cell lineage-specific factor paired box protein 5 (PAX5) at the immunoglobulin heavy chain (IgH) locus during B-cell development [52,58].

Similar to the architectural changes observed at the TCRα/TCRδ locus during thymocyte development [50], distinct genomic conformations have been observed by confocal microscopy at the TCRβ locus during DN3a to DP differentiation [51] (Figure 6b). In DN3a thymocytes, the entire TCRβ locus presents a completely compacted configuration, bringing all Vβ gene segments into spatial proximity to the 25-kb TCRβ D-J region to assure a diverse TCRβ repertoire [51,59,60,61,62,63]. This locus architecture is thought to be mediated by CTCF/cohesin binding to CBSs associated with half of the Vβ gene segments (such as the A, B, and C sites), two intergenic sites located upstream of the Dβ gene segments (5′PC and D sites), and another one (E site) located downstream of the TCRβ enhancer (Eβ). CBSs associated to the Vβ gene segments are all oriented convergently toward the 5′PC, D, and E sites [47,63], allowing CTCF-CTCF looping formation. These loops bring the Vβ gene segments into the vicinity of the DβJβ region via locus contraction. In DP thymocytes, when the TCRβ gene has been rearranged and the corresponding TCRβ successfully selected, the most distal Vβ region becomes spatially segregated from the D-J region, contributing to the allelic exclusion of this locus [51]. The inhibition of further TCRβ gene rearrangements in DP thymocytes is also associated with changes in Vβ chromatin structure, which inhibits the recruitment of RAG1 and RAG2 to the RSSs [64,65].

In addition to these mechanisms in DP thymocytes, TCRβ gene allelic exclusion in DN3a thymocytes is assured via frequent stochastic association of both alleles with the nuclear lamina and pericentromeric heterochromatin, thereby inhibiting Vβ-DβJβ recombination [33,66,67]. Further, recent data have demonstrated that TCRβ gene allelic exclusion in DN3a thymocytes also occurs through suboptimal RSSs at the Vβ gene segments to limit synchronous Vβ-DβJβ rearrangements [68]. The strong peripheral nuclear localization of the TCRβ locus in DN3a and DP thymocytes contrasts with that of the TCRα/TCRδ locus, which moves from a predominant peripheral localization in pro-B cells, which rearrange the IgH locus but not the TCRα/TCRδ locus, to a more central localization in DN3a and DP thymocytes [33,51,69]. This data indicate that the TCRα/TCRδ locus is associated to peripheral nuclear locations, such as nuclear lamina and peripheral heterochromatin, in precursors destined to give rise to non-T cell lineages. At present, it is unclear whether the developmentally regulated-CTCF/cohesin-dependent interaction networks formed at the Vα/δ region are involved in the movement of the TCRα/TCRδ locus from the nuclear periphery to a more nuclear central position [33].

### 4.2. Topological Gene Changes at the TCRδ, TCRα, and TCRβ (D)-J Regions

In addition to the V regions, the D-J regions of the TCRα/TCRδ and TCRβ loci also undergo considerable conformational changes during thymocyte development [28,61] (Figure 6). These regions have been defined as recombination centers (RCs) due to the high corecruitment of RAG1 and RAG2 to actively transcribed D and J gene segments [62]. The TCR RC boundaries have been defined by the identification of CBSs where CTCF and cohesin can simultaneously bind and by the detection of interactions between regulatory regions via 3C-derived techniques [28,47,53,61]. The CTCF/cohesin- and enhancer-promoter-mediated loops detected at the Dδ-Jδ and Dβ-Jβ regions in DN3a thymocytes and the Jα regions in DP thymocytes define the TCRδ, TCRβ, and TCRα RCs, respectively [28,47,61,62,70].

The TCRδ RC is defined by an 80-kb loop between two intergenic CBSs (INT1 and INT2), which are separated by 5 kb and located between the *Trdv-2* and *Trdv4* gene segments, and a convergent CBS present at the TEAp, which is located between the TCRδ and TCRα genes [28,47,71] (Figure 6a). This INT1/2-TEAp loop favors the usage of distal Vδ and Vα/δ gene segments in the VδDδJδ rearrangements [71]. In the absence of this loop in INT1/2^-/-^ DN3a thymocytes, an alternative loop is formed between a CBS upstream of the *Trdv2-2* gene segment and TEAp, resulting in almost exclusive usage of the *Trdv2-2* gene segment in VδDδJδ rearrangements. Hence, the INT1/2-TEAp loop formed in DN3a thymocytes serves to diversify the TCRδ repertoire.

The TCRα RC is defined by a 90-kb loop established between TEAp and Eα [28,47,52,53,70] (Figure 6a). This RC interacts with the proximal Vα region through additional chromatin loops to form the 525-kb chromatin hub found in DP thymocytes [28,47,52,53]. Formation of this RC favors the processive VαJα rearrangements by using 5′-to-3′ Jα gene segments [52,54,55,56].

The diverse TCRδ gene rearrangements that result from formation of the INT1/2-TEAp loop in DN3a thymocytes redirect the processive VαJα rearrangement program in DP thymocytes [56,71]. Therefore, the combination of chromatin loops formed during thymocyte development increases the repertoire of the TCRδ and TCRα genes.

The TCRβ RC is defined by Eβ-Dβ1 promoter and Eβ-Dβ2 promoter loops formed in a 25-kb region [61,72], emphasizing Eβ as the crucial element in RC establishment (Figure 6b). In contrast with the TCRδ and TCRα RCs, where CTCF and cohesin bind to convergent CBSs and play a functional role in RC formation [52,53,71], no convergently oriented CBSs are found within the TCRβ RC (5′PC, D, and E sites have the same orientation) [47,63]. However, tandem CBSs flanking the TCRβ RC can create coiled loops that could contribute to the formation and stabilization of this RC as well as to the configuration of specific loops that facilitate RSS synapsis and recombination. Although convergent and tandem CBSs fold the intervening chromatin differently [73], the fact that all TCR gene RCs are flanked by CBSs supports an important role for these sites in the dynamics of RC formation.

## 5. TCR Enhancers and Function

Developmental regulation of gene transcription in higher eukaryotes is controlled by enhancers located distantly from their regulated promoters [74]. As mentioned before, TCR enhancers are important components of the RC chromatin structure [54,72,75], participating in their formation through enhancer-promoter-mediated loops, as is the case at the TCRβ gene in DN3a thymocytes and at the TCRα gene in DP thymocytes [52,72]. However, these enhancers (Eβ and Eα, respectively) are not required for the global 3D locus architectural configurations affecting the V regions or the gene movements between different nuclear locations that occur during thymocyte development [50,52,60]. This suggests the existence of two levels of chromatin compaction at the TCR loci during thymocyte development. First, enhancer-independent locus compaction occurs that depends on CTCF/cohesin and additional factor binding to the V regions, bridging considerable genomic distances [47]. This gene configuration precedes or is coincidental with an enhancer-dependent level of compaction established by enhancer-promoter contacts (and which could also be facilitated by CTCF/cohesin-mediated loops) to create an RC that promotes germline transcription and V(D)J recombination at the appropriate thymocyte stage [23,36,52,60,70,72,75,76,77,78,79,80]. Within the D-J regions, activation of TCR enhancers controls RC formation through interactions with specific D and/or J gene segment promoters. This induces chromatin changes and germline transcription that drives V(D)J recombination through the recruitment of RAG1 to the exposed RSSs that flank the D and/or J gene segment and RAG2 to trimethylated histone H3 lysine 4 residues present in transcriptionally active genomic regions [52,60,62,70,80,81,82,83]. Hence, the transition from an unrearranged to a rearranged TCR gene configuration occurs in an enhancer-, promoter- and transcription-dependent manner by promoting the cobinding of RAG1 and RAG2 to the gene segments present within a given RC [70].

Precedents for the requirement of multiple enhancers for V(D)J recombination control have been found in other antigen receptor loci, such as the IgH and Igκ loci. These enhancers all have complementary nonoverlapping functions in the formation of specific chromatin loops to control V(D)J recombination [84,85,86]. However, with the exception of the murine TCRγ locus, where two enhancers collaborate in the regulation of germline transcription and VγJγ recombination [87], a unique enhancer located in the proximity of the C region is required to activate germline transcription and V(D)J recombination at all the other murine and human TCR loci [23,36] (Figure 4).

At the TCRα/TCRδ locus, Eα is the only known regulatory TCRα gene enhancer and is located a few kb downstream of Cα, while the TCRδ gene enhancer (Eδ) is located in the murine Jδ2-Cδ intron or the human Jδ3-Cδ intron [88,89,90,91]. The murine TCRγ locus contains several cooperating enhancers, including a TCRγ gene enhancer (Eγ) positioned downstream of each Cγ region and another enhancer within the γ1 cluster, denoted as HsA, whereas the human TCRγ locus contains one unique Eγ located downstream of Cγ2 [92,93,94,95,96]. Eβ is the unique TCRβ gene enhancer and is located downstream of Cβ2 [97,98,99]. All these enhancers are essential for the RC chromatin changes and germline transcription that trigger gene rearrangements at their respective loci [87,100,101,102,103] and for TCR chain expression and generation of αβ and γδ T lymphocytes.

## 6. Signaling-Dependent Control of TCR Enhancers during Thymocyte Development

Enhancer function is responsible not only for activation of TCR gene expression but also for TCR gene silencing during thymocyte development. Specifically, enhancers are responsible for TCRγ, TCRδ, and TCRβ gene expression in DN3a thymocytes, as well as for TCRβ and TCRα gene expression and TCRγ and TCRδ silencing in DP thymocytes [13,44,104]. The correct regulation of the TCR enhancers during development is important for thymocyte maturation and generation of αβ and γδ T lymphocytes. Premature activation of Eα prior to β-selection would inhibit the generation of γδ T lymphocytes through the deletion of the TCRδ gene as a consequence of early VαJα recombination. Although minimal premature VαJα rearrangements have been detected in the context of Eα^-/-^ mice [100,105] or in the absence of pre-TCR signaling [106], the rearrangements that do occur result from the activity of Eδ rather than from the early activation of Eα in DN3a thymocytes [105]. In addition, the expression of a premature TCRαβ would negatively affect the proper development of αβ T lymphocytes because TCRαβ signals do not efficiently trigger β-selection compared to those derived from the expression of the pre-TCR [107]. Furthermore, early expression of the TCRα chain can result in the generation of TCRαγ complexes, impairing normal αβ T cell development [46]. Specific mechanisms exist to circumvent premature activation of Eα, premature TCRαβ expression, assembly of TCRαγ in DN3a thymocytes, and inhibition of γδ T lymphocyte generation [108]. Furthermore, silencing of the TCRγ and TCRδ genes in DP thymocytes and αβ T lymphocytes through Eγ and Eδ inactivation is important to avoid expression of TCRγ and TCRδ chains that would interfere with the correct assembly of TCRαβ complexes in these cells [13,44,46,104].

**Figure 7:**
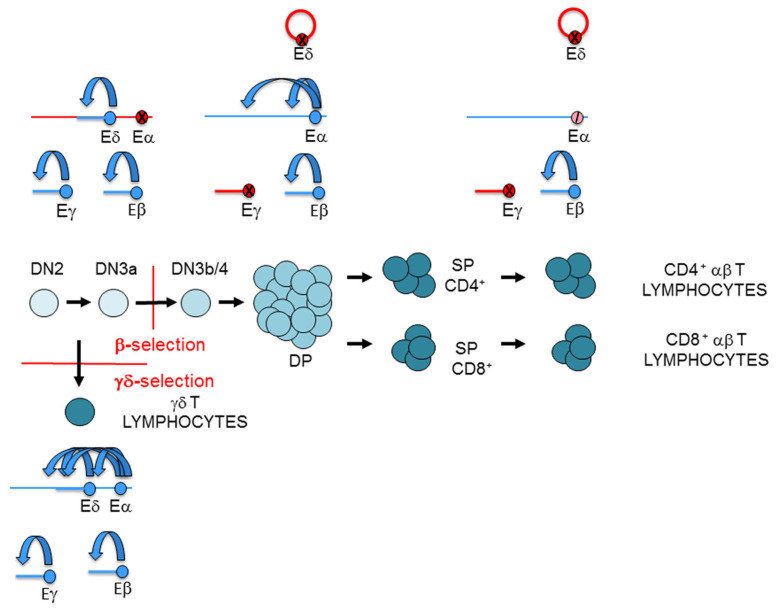
Representation of the T-cell receptor (TCR) enhancer activity during thymocyte development: TCRα enhancer (Eα), TCRβ enhancer (Eβ), TCRγ enhancer (Eγ), and TCRδ enhancer (Eδ). T-cell maturation in the thymus is depicted. Active enhancers and genes are represented in blue, inactive enhancers and genes are represented in red, and an inhibited enhancer is represented as a crossed pink circle. In double-negative 3a (DN3a) thymocytes, Eβ, Eγ, and Eδ are active, whereas Eα is inactive. In double-positive (DP) thymocytes, Eγ and Eδ are inactive, whereas Eβ and Eα are active. In αβ T lymphocyte, Eγ and Eδ are inactive, Eβ remains active, and Eα is strongly inhibited. In γδ T lymphocyte, the four enhancers are active. In consequence, the TCRγ and TCRδ genes are expressed in DN3a thymocytes and γδ T lymphocytes, but not in DP thymocytes and αβ T lymphocytes. In contrast, the TCRα gene is not expressed in DN3a thymocytes, but it is expressed in DP thymocytes. Despite being Eα strongly inhibited in αβ T lymphocytes, the TCRα gene is highly expressed. In accordance with strong Eβ activity in DN3a thymocytes and in αβ and γδ T lymphocytes, TCRβ gene transcription is detected in all these cells.

**Figure 8:**
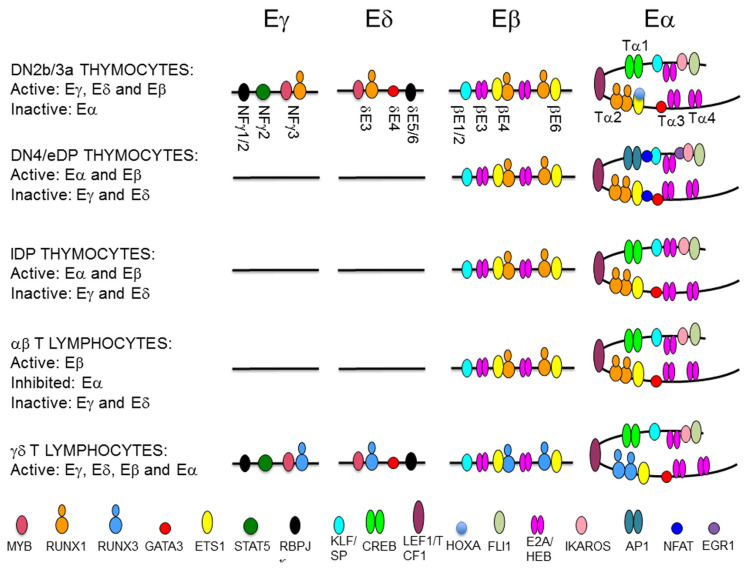
Representation of transcription factor (TF) binding to T-cell receptor (TCR) enhancers during thymocyte development. The diagram depicts the TF that are recruited to the TCRγ enhancer (Eγ), the TCRδ enhancer (Eδ), the TCRβ enhancer (Eβ), and the TCRα enhancer (Eα) during T cell maturation. The location of defined enhancer elements are indicated. TFs are represented as colored ovals as indicated.

The dependence on TCR enhancers for specific signaling provokes their activation or inactivation during thymocyte development (Figure 7), through the recruitment of specific transcription factors (TFs) (Figure 8). Pre-TCR signaling is critical to control the developmental switch formed by Eγ/Eδ and Eα during thymocyte maturation, with active Eγ/Eδ and inactive Eα in DN3a thymocytes, and inactive Eγ/Eδ and active Eα in DP thymocytes [13,44,109]. Notch signaling activates Eγ and Eδ in DN3a thymocytes, whereas pre-TCR-induced *Notch1* gene silencing inhibits these enhancers and activates Eα function in DP thymocytes [6,13,110] (Figure 7). Specifically, Eγ and Eδ function depends on specific Notch-dependent binding of RUNX1 and MYB to essential enhancer sites in DN3a thymocytes [13,44,104,111,112,113,114,115]. Hence, pre-TCR-regulated expression of *Notch1* during β-selection [110] constitutes a key point in the regulation of Eγ and Eδ activity, resulting in enhancer inactivation through the dissociation of RUNX1 and MYB from the composite sites to silence TCRγ and TCRδ gene expression in DP thymocytes [13,44,104]. In addition, IL-7R signaling in DN3a thymocytes plays a crucial role in Eγ and HsA function, via the recruitment of STAT5 to essential binding sites, opening TCRγ gene chromatin, and activating germline transcription and VγJγ recombination [38,42,43]. Hence, Eδ and Eγ silencing during DN3a to DP thymocyte maturation occurs through pre-TCR-dependent RUNX1, MYB, and STAT5 dissociation from enhancers as a consequence of Notch and IL-7R signaling termination, thereby constituting the molecular mechanism of TCRγ and TCRδ gene transcriptional silencing during β-selection [13,44,104]. In addition, as a consequence of RUNX1 dissociation, GATA3 is dissociated from Eδ during the transition from DN3a to DP thymocytes [44,113].

In DN3a thymocytes, Eα exists as a poised enhancer constitutively bound by essential TFs, such as CREB, LEF1/TCF1, RUNX1, and ETS1, in addition to GATA3, E2A, HEB, FLI1, SP1, and IKAROS, but it remains inactive due to the recruitment of HOXA TFs through their interaction with ETS1 [44,108,109,116,117,118,119,120,121]. In agreement with its poised state in the early stages of thymic maturation, Eα is in an open chromatin configuration, fully demethylated, and enriched in H3K4me1 histone marks in human DN thymocytes [108]. The activation of Eα during β-selection results in the recruitment of pre-TCR-induced TFs, such as NFAT, EGR1, and AP1, and the termination of binding of repressive TFs, such as HOXA, to a preassembled enhancer [108,109]. Eα activation results in increased H3K27ac and H3K4me3, which are associated with active enhancers, and the induction of enhancer RNA [108,122]. In contrast with the dynamic and opposite regulation of Eγ/Eδ and Eα, Eβ is constitutively active during DN3a to DP thymocyte development [123] (Figure 7). Eβ depends on the specific binding of RUNX1 and ETS1 to essential binding sites in DN3a and DP thymocytes [119,124,125]. A representation of the binding of key TFs to the TCR enhancer during T lymphocyte development is shown in Figure 8.

In spite of their different mechanisms of activation, the requirement of RUNX1 binding is common across the TCR enhancers. RUNX1 activity depends on its specific interactions with other TFs that bind to DNA in the vicinity of the RUNX site, as has been found for MYB and ETS1 at the TCR enhancers. RUNX1 cooperates with MYB in the regulation of Eγ and Eδ by binding to a composite MYB-RUNX site and with ETS1 in the regulation of Eα and Eβ by binding to composite RUNX-ETS sites (one in Eα and two in Eβ) [13,111,112,116,120,124,125,126]. RUNX1 binds independently of MYB to Eδ in vitro, acting as a structural TF, and facilitates the recruitment of MYB and GATA3 to this enhancer in vivo [112,113]. Similar RUNX1-dependent MYB binding is expected to play a role in the regulation of Eγ. In contrast, RUNX1 and ETS1 bind cooperatively to composite sites in Eβ and Eα in vitro and in vivo [116,119,120,124,125]. Studies with mutant versions of Eδ and Eβ indicate that different factors play functional and structural roles in the MYB-RUNX1 and RUNX1-ETS1 pairs [113,124]. At the MYB-RUNX site, MYB is the functional TF for Eδ activation, while RUNX1 acts as a structural factor that facilitates the recruitment of MYB to the enhancer chromatin. However, at the composite RUNX-ETS site, RUNX1 is the functional TF for Eβ function, whereas ETS1 enhances binding of the complex to DNA through physical interactions with RUNX1 [116,124]. In fact, induced RUNX1 binding to Eβ in the absence of ETS1 binding is sufficient for long-distance enhancer-promoter looping within the TCRβ RC to activate nucleosome clearance and germline transcription [124]. In contrast with the stable binding of RUNX1 and ETS1 to their composite sites at Eα and Eβ during β-selection [44,108,118,123,125], the recruitment of RUNX1 and MYB to Eγ and Eδ is less stable and is dependent on Notch signaling [13]. These different properties of the MYB-RUNX1 and RUNX1-ETS1 complexes bound to their respective composite sites permit the dynamic regulation of TCRγ and TCRδ gene expression and ensures the stable expression of TCRβ during β-selection [44,45]. Despite the stable binding of RUNX1 and ETS1 to Eα in DN3a thymocytes, this enhancer can be inhibited via the recruitment of HOXA TFs through the ETS1/RUNX1 complex [108].

TCR gene transcription at rearranged genes depends on enhancer-dependent activation of the recombined V gene segment. However, the relevant enhancers or enhancer sequences required for transcribing the rearranged TCR gene could be different from those previously required for germline transcription and V(D)J recombination. That is the case for the transcriptional regulation of the rearranged TCRβ and TCRα/TCRδ loci in mature T lymphocytes [100,127]. At the TCRβ locus, different Eβ elements regulate germline transcription and V(D)J recombination in DN3a thymocytes versus transcription of the rearranged gene in αβ T lymphocytes [123]. Furthermore, different RUNX complexes are required for Eβ activation at these cell stages, illustrating the distinct roles played by this TF in the initiation and the maintenance of enhancer function [128]. At the TCRδ gene, Eδ is involved in germline Dδ and Jδ transcription and VδDδJδ recombination in DN3a thymocytes, but it is not required for transcription of the rearranged TCRδ gene in γδ T lymphocytes [101], with Eα serving as the relevant *cis*-regulatory region in this context [100] (Figure 7). The inverse ratio of *Runx1* and *Runx3* transcripts present in DN3a thymocytes and γδ T lymphocytes suggests that an interchange between RUNX1 and RUNX3 might occur to guarantee enhancer function for TCRγδ expression at the latter cells [129] (Figure 8). In the case of the TCRα gene, Eα is the relevant enhancer required for germline transcription and primary VαJα recombination [100]; however, it is strongly inhibited by 85% in SP thymocytes and αβ T lymphocytes [127] (Figure 7). Therefore, pre-TCR signaling during β-selection and TCRαβ signaling during positive-selection have opposite effects on Eα activity. Eα downmodulation in αβ T lymphocytes is associated with loss of E2A and HEB recruitment [127] (Figure 8). Although the strong Eα downmodulation observed in αβ T lymphocytes suggests the existence of an Eα-independent mechanism for transcribing the rearranged TCRα gene, the function of this enhancer in αβ T lymphocytes is not clear at present. The strong inhibition of Eα activity in αβ T lymphocytes contrasts with its essential role in the transcription and expression of the rearranged TCRδ in γδ T lymphocytes, where the TCRδ gene remains in *cis* with the enhancer [100].

## 7. Concluding Remarks

TCR gene expression is a precisely controlled process in a cell-type and stage-specific manner through the interaction between cis-regulatory elements, such as CBSs, enhancers, and promoters, and their associated factors. This complex process needs to be orchestrated through highly regulated mechanisms, which include architectural TCR gene changes and an exquisite control of the enhancers at the different developmental cell stages. In this context, the 3D organization of the TCR genome provides insulated loop structures to restrict enhancer and promoter function. Although CTCF and cohesin factors are crucial in controlling the conformational dynamics observed at the TCR loci, additional unknown specific T-cell factors are clearly involved. Future experiments will identify the factors and signaling pathways involved in the conformational changes of TCR loci during thymocyte development. Another important aspect to understand the molecular mechanisms involved in TCR gene expression includes the study of enhancer function during thymocyte development. Notch-dependent recruitment of RUNX1 and MYB is crucial for Eγ and Eδ activity during thymocyte development and, hence, for TCRγ and TCRδ gene expression. Whether the regulation of RUNX1 and MYB recruitment constitutes a general mechanism for Notch-dependent regulation of gene expression during β-selection is an open question. In addition, similarly to the parallel regulation of Eδ and Eγ by Notch signaling, it is possible that IL-7R might play a similar role in Eδ function and TCRδ gene expression as it does for the regulation of the Eγ and TCRγ genes. These two signaling pathways collaborate in the generation of leukemia [130,131], and it is intriguing to consider whether they may do so through the synergistic regulation of aberrant oncogene translocations through Eδ- and Eγ-dependent illegitimate V(D)J recombination at the TCRδ or TCRγ genes. In addition, the molecular mechanisms involved in the fine-tuned regulation of Eα activity and TCRα gene expression during T-cell development, which is inhibited in DN3a thymocytes, fully active in DP thymocytes, and strongly downmodulated in αβ T lymphocytes, are important questions to address in future investigations. These include the mechanisms by which TCRα gene silencing occurs in DN3a thymocytes and the rearranged TCRα gene is efficiently expressed in αβ T lymphocytes. Future efforts should identify and evaluate how TCR enhancers establish long-distance interactions with promoters and/or other still unknown genomic regions in the context of the unrearranged and rearranged TCR genes in thymocytes and mature αβ and γδ T lymphocytes. Future work will also investigate the detailed molecular mechanisms involved in enhancer function during T-cell development through their capacity to form phase-separated condensates that might contribute to the formation of the 3D genome structure.

## Abbreviations

3C	Chromosomal conformation capture
3D	Three dimensional
APC	Antigen presenting cell
β2m	>β2 microglobulin
bp	Base pairs
C	TCR gene constant region
Cα	TCRα gene constant region
Cβ	TCRβ gene constant region
Cδ	TCRδ gene constant region
Cγ	TCRγ gene constant region
CBS	CTCF binding site
CDR	Complementarity-determining region
CTCF	CCCTC-binding factor
D	Diversity (gene segment)
DN	Double-negative
DP	Double-positive
Eα	TCRα enhancer
Eβ	TCRβ enhancer
Eδ	TCRδ enhancer
eDP	Early DP
Eγ	TCRγ enhancer
HSC	Hematopoietic stem cell
IgH	Immunoglobulin heavy chain
IL-7R	Interleukin 7 receptor
ISP	Immature Simple positive
J	Joining (gene segment)
lDP	Late DP
MHC	Major histocompatibility complex
MHC-I	MHC class I
MHC-II	MHC class II
PAX5	Paired box protein 5
pTα	Pre-T α
RAG	Recombination activating gene
RC	Recombination center
RSS	Recombination signal sequence
SP	Simple positive
TCR	T-cell receptor
TEA	T early α exon exon
TEAp	TEA promoter
TF	Transcription factor
V	Variable (gene segment)
YY1	Yin-Yang protein
